# Predictors of medical student interest in Indigenous health learning and clinical practice: a Canadian case study

**DOI:** 10.1186/s12909-018-1401-1

**Published:** 2018-12-14

**Authors:** Sharon Yeung, Amy Bombay, Chad Walker, Jeff Denis, Debbie Martin, Paul Sylvestre, Heather Castleden

**Affiliations:** 1 Department of Public Health Sciences, Queen’s University, Kingston, Ontario Canada; 2 School of Medicine, Queen’s University, Kingston, Ontario Canada; 3 Department of Psychiatry and School of Nursing, Dalhousie University, Halifax, Nova Scotia Canada; 4 Department of Geography and Planning, Queen’s University, Kingston, Ontario Canada; 5 Department of Sociology, McMaster University, Hamilton, Ontario Canada; 6 School of Health and Human Performance, Dalhousie University, Halifax, Nova Scotia Canada

**Keywords:** Indigenous health, Cultural safety, Experiential learning, Attitude change, Racism

## Abstract

**Background:**

Including content on Indigenous health in medical school curricula has become a widely-acknowledged prerequisite to reducing the health disparities experienced by Indigenous peoples in Canada. However, little is known about what levels of awareness and interest medical students have about Indigenous peoples when they enter medical school. Additionally, it is unclear whether current Indigenous health curricula ultimately improve students’ beliefs and behaviours.

**Methods:**

A total of 129 students completed a 43-item questionnaire that was sent to three cohorts of first-year medical students (in 2013, 2014, 2015) at one undergraduate medical school in Canada. This survey included items to evaluate students’ sociopolitical attitudes towards Indigenous people, knowledge of colonization and its links to Indigenous health inequities, knowledge of Indigenous health inequities, and self-rated educational preparedness to work with Indigenous patients. The survey also assessed students’ perceived importance of learning about Indigenous peoples in medical school, and their interest in working in an Indigenous community, which were examined as outcomes. Using principal component analysis, survey items were grouped into five independent factors and outcomes were modelled using staged multivariate regression analyses.

**Results:**

Generally, students reported strong interest in Indigenous health but did not believe themselves adequately educated or prepared to work in an Indigenous community. When controlling for age and gender, the strongest predictors of perceived importance of learning about Indigenous health were positive sociopolitical attitudes about Indigenous peoples and knowledge about colonization and its links to Indigenous health inequities. Significant predictors for interest in working in an Indigenous community were positive sociopolitical attitudes about Indigenous peoples. Knowledge about Indigenous health inequities was negatively associated with interest in working in an Indigenous community.

**Conclusions:**

Students’ positive sociopolitical attitudes about Indigenous peoples is the strongest predictor of both perceived importance of learning about Indigenous health and interest in working in Indigenous communities. In addition to teaching students about the links between colonization, health inequities and other knowledge-based concepts, medical educators must consider the importance of attitude change in designing Indigenous health curricula and include opportunities for experiential learning to shape students’ future behaviours and ultimately improve physician relationships with Indigenous patients.

**Electronic supplementary material:**

The online version of this article (10.1186/s12909-018-1401-1) contains supplementary material, which is available to authorized users.

## Background

In Canada and around the world, Indigenous peoples experience significant health inequities compared to non-Indigenous peoples. [1–3] These persistent inequities are consequences of settler-colonialism, perpetuated by settler^1^ social systems and institutions that actively efface and misrepresent Indigenous realities and lifeways. [1, 4, 5] Indigenous peoples’ health has been, and continues to be, harmed by individual, structural, and institutional racism that is perpetuated by health care systems and individual providers. [3, 6–8] To help address these health inequities, it has been generally acknowledged that cultural safety programming must be embedded in the education of health care professionals. The importance of education has been reiterated by the Truth and Reconciliation Commission of Canada; the final report in 2015 recommended that students in medical and nursing schools be mandated to take a comprehensive course on Indigenous health. [9] In order to increase our understanding of how Indigenous health curricula can be improved based on a target audience, this study explored first-year students’ existing knowledge and attitudes towards Indigenous peoples at one Canadian medical school. This study also sought to identify the factors most strongly predictive of two outcomes related to the intentions of the Indigenous health curriculum: students’ perceived importance of further learning about Indigenous health, and student interest in working in Indigenous communities, positing that the predictive factors of these outcomes may be essential elements to include in cultural safety training.

In pedagogy, the triadic teaching of knowledge, skills, and attitudes is traditionally applied to specific competencies. To date, most medical teaching pertaining to cultural safety emphasizes delivery of knowledge, at the expense of encouraging students to reflect upon their attitudes and developing their practical skills. [10] Although the study of medical students’ specific knowledge of Indigenous health topics is sparse, Zhou et al. (2011) [11] recently identified that medical students tend to be aware of various Indigenous health issues, but their understanding of the effect of sociocultural and economic factors on Indigenous peoples’ health is more limited. Furthermore, their knowledge may be rooted in stereotypical conceptions and portrayals of Indigenous peoples, in part borne from negative views encountered through the media, as well as throughout their education and in medical school from peers and teachers. [12–15] The delivery of relevant knowledge bases, particularly pertaining to the social determinants of health, has been shown to positively impact the comfort of medical interns with discussing social issues with their patients and their ability to self-reflect [16, 17], but there are also limitations to the impact of knowledge alone. In this regard, Beagan (2003) [18] reported that medical students at one Canadian University believed that learning about social and cultural issues was “all very nice to talk about in theory, but ultimately it makes no difference [on the wards].” More recently, Bullen et al. [15] suggested that positive attitudes are more significantly associated with self-perceived preparedness to work with Indigenous Australians, further bringing into question the adequacy of current knowledge-focused cultural safety curricula.

Among the handful of studies that have investigated medical students’ perspectives on Indigenous health, results indicate that many have substantial interest in learning more and may even consider working in Indigenous contexts, but largely feel unprepared to do so. [11, 12, 19, 20] These results have been replicated at the level of post-graduate trainees, in both family medicine and obstetrics and gynecology. [11, 19, 20] Various cultural safety initiatives have further identified that among students, interest in Indigenous peoples’ cultures, culturally safe practice, and advocacy work can be nurtured through appropriate programming. [21–23] For example, the Aboriginal Cultural Safety Initiative that was developed by Anishnawbe Health Toronto for college and university students throughout Ontario showed promise as a program that increased student interest in Indigenous health following a curriculum on social determinants on Indigenous health, Indigenous concepts of health and wellness, and impacts of colonial policies. [22] Similarly, Zhou et al. [11] found that a three-hour Indigenous health seminar delivered to medical students on similar content showed some promise in increasing interest in working in First Nations, Métis, and Inuit communities in Canada, in addition to impacting attitudes and feelings of preparedness to work with Indigenous peoples.

Despite advances in the integration of Indigenous health content into medical school curricula, it remains unclear what an ideal Indigenous health curriculum that further increases student interest in Indigenous health and their preparedness to work in Indigenous communities should resemble. Consequently, a robust standard for medical education on Indigenous health topics remains an unmet need. In the current study, we contribute to addressing this knowledge gap by examining first-year medical students’ sociopolitical attitudes towards Indigenous people, knowledge of colonization and its links to health inequities, knowledge of Indigenous health inequities, and self-rated educational preparedness to work with Indigenous patients. Further, we examine students’ perceived importance of learning about Indigenous peoples in medical school, and interest in working in an Indigenous community in the future. Given the diversity of student demographics, admissions processes, and teaching methods across medical schools in Canada, this study is only an exploratory investigation of the perspectives of students at a single academic institution, but lessons learned are broadly applicable to medical education on Indigenous health. As the demographic of this study also represents the intended audience for cultural safety training in medical schools, the results also help to establish an understanding of entry-level perspectives (i.e. prior to the delivery of medical school curricula) and to shed some light on the curricular content that may be most impactful in shaping students’ beliefs and interests, which are among the target outcomes of cultural safety training and programming.

## Methods

### Participants and procedures

A self-report questionnaire was administered to 129 first-year medical students from an academic institution in Canada during the Fall term of three consecutive years: 2013, 2014, and 2015. (Table 1) With permission from the Dean, electronic invitations to participate were disseminated via class distribution lists and questionnaires were completed via an online form; although it would have been preferable for students to complete the questionnaire during in-class time to garner a higher response rate, this was not permitted. Ethical clearance was obtained from associated Research Ethics Boards and informed consent was provided by all participants.

**Table 1:** Characteristics of study sample

Characteristics	Groups	N(%)
Gender	Male	52 (40.3)
	Female	76 (58.9)
	Other	1 (0.8)
Age	18–24	68 (52.7)
	25–34	59 (45.7)
	35+	2 (1.6)
Year of survey completion	2013	39 (30.2)
	2014	53 (45.7)
	2015	37 (28.7)

In addition to questions gathering demographic information, the measures used in the questionnaire were created through the adaptation of Likert scale items from four previous surveys, one of which (Zhou et al. [11]) investigated medical trainees’ willingness and preparedness to work in Indigenous communities. The other sources (Environics Institute [24];Denis [25]; Ipsos-Reid [26]) examined racism and prejudice among the wider Canadian public. These surveys were adapted for this study with input from an Indigenous Advisory Committee (IAC) comprised of individuals with expertise in Indigenous health, survey administration, program evaluation, and medical school curricula. The final study instrument is appended as an Additional file 1.

### Measures

This study had two dependent variables of interest that were chosen based on input from the IAC and because of their relevance to the intended attitudinal and behavioural outcomes of cultural safety training. [27–29] First, *‘Perceived importance of Indigenous content in curricula’* was assessed using the following four items: [1] It is valuable for physicians to be educated on Indigenous social issues, [2] It is valuable for physicians to be educated on Indigenous health issues, [3] Conventional (Western) physicians should be aware of traditional medicine(s) that their patients may be using, and [4] The clinical curriculum should incorporate a rotation in an Indigenous community. These items were amalgamated to represent this overarching construct on the basis of principal component analysis (PCA), which identified primary loading of these four items onto the same factor, with minimal cross-loadings. The second dependent variable was ‘*Interest in working in an Indigenous community*’ and was assessed using one item that asked students whether they would consider working in a First Nations, Inuit, or Métis community. This dependent variable was identified at face value as an outcome of interest. All items were scored using a four-point scale: ‘Strongly disagree’ [1], ‘Disagree’ [2], ‘Agree’ [3], ‘Strongly agree’ [4]. There was a fifth option of ‘I don’t know’. Responses of ‘I don’t know’ were excluded from analysis.

There were four independent variables of interest: [1] *‘Sociopolitical attitudes about Indigenous peoples*’; [2] *‘Knowledge of colonization and its link to Indigenous health inequities’*; [3] *‘Knowledge of Indigenous health inequities*’; and [4] *‘Self-rated educational preparedness to work with Indigenous patients’*. Each of these variables were constructed by amalgamating the scores of various items of the questionnaire as indicated by the results of an exploratory PCA of all survey questions. This exploratory PCA generated a five-factor solution (Table 2) that captured 64.47% of total variance in responses, of which four factors were used as the independent variables listed above. PCA was conducted with a varimax rotation and Kaiser normalization, and items with loadings < 0.3 were suppressed. Factor scores were constructed by averaging the constituent variables of each factor and each factor was labeled as an independent variable as inferred by the researchers. Internal consistency within each factor was adequate (‘*Sociopolitical attitudes about Indigenous peoples*’: 0.77; ‘*Knowledge of colonization and its link to health inequities*’: 0.81; ‘*Knowledge of Indigenous health inequities’*: 0.82; ‘*Self-rated educational preparedness to work with Indigenous patients*’: 0.82). Both outcomes were significantly associated with Factors 1 through 4 (Pearson coefficients 0.290–0.599, *p* < 0.05), with the exception of ‘*Knowledge of Indigenous health inequities’*, which was not significantly correlated with the dependent variable ‘*Perceived importance of Indigenous content in curricula’*. Factors were not strongly correlated with one another (Pearson coefficients 0.00–0.48), confirming the constructs represent distinct concepts. (Table 2).

**Table 2:** Results of Principal Components Analysis and descriptive statistics of items used to assess dependent variables (*N* = 129)

	Component Loading ^b^	Mean out of 4 (SD)	Strongly disagree N(%)	Somewhat disagree N(%)	Somewhat agree N(%)	Strongly agree N(%)	I don’t know N(%)
*Perceived importance of Indigenous* *content in curricula (α = 0.805)* ^a^		3.48 (0.63)					
Conventional (i.e. western) physicians should be aware of traditional medicine(s) that their patients may be using.	0.694	3.65 (0.638)	3 (2.3)	2 (1.6)	31 (24.0)	89 (69.0)	4 (3.1)
It is valuable to me as a physician/in-training to be educated on social issues concerning Aboriginal peoples.	0.884	3.52 (0.768)	5 (3.9)	6 (4.7)	33 (25.6)	81 (62.8)	4 (3.1)
It is valuable to me as a physician/in-training to be educated on the health issues concerning Aboriginal peoples	0.877	3.60 (0.718)	4 (3.1)	5 (3.9)	29 (22.5)	88 (68.2)	3 (2.3)
The clinical curriculum should incorporate a rotation in an Aboriginal community	0.630	3.16 (0.974)	12 (9.3)	12 (9.3)	40 (31.0)	55 (42.6)	10 (7.8)
*Interest in working in an Indigenous community*							
I would consider working in a First Nations, Métis, and/or Inuit community	N/A	2.68 (1.11)	21 (16.3)	18 (14.0)	32 (24.8)	28(21.7)	30 (23.3)

^a^ Cronbach’s alpha for the subscale

^b^ Component loadings < 0.4 suppressed

### Analyses

In addition to descriptive analyses, two regression analyses were performed to assess the predictors of the two dependent variables of interest. First, linear regression was used to model students’ perceived importance of Indigenous health in curricula (Factor 3). Statistical tests were two-tailed, with a significance level of 0.05. Second, logistic regression was used to model student interest in working in an Indigenous community. This outcome variable was binary (agree/disagree) and was represented by the response to a single item on the questionnaire (“I would consider working in a First Nations, Métis, and/or Inuit community”). All statistical analyses were performed using SPSS v.24.0 (IBM Corp., Armonk, NY, USA).

## Results

### Descriptive statistics

In total, 129 first-year students responded to the survey (39 from 2013, 53 from 2014, 37 from 2015). Just over half of the participants were female (76, 58.9%), consistent with national medical student demographics, and the majority were aged 18–24 (68, 52.7%) or 25–34 (59, 45.7%). With regard to self-identified ethnicity, the majority of participants (103, 79.8%) indicated ‘Canadian’ and only 6 participants (4.7%) reported Indigenous ancestry.^2^ (Table 1).

### Dependent variables: (Table 2)

#### ‘Perceived importance of Indigenous content in curricula’

Overall, participants agreed that it is valuable for physicians to be educated on social issues concerning Indigenous peoples (114, 88.4%) and health issues concerning Indigenous peoples (117, 90.7%); over half of the participants strongly agreeing with these statements (81, 62.8% for social issues; 88, 68.2% for health issues). The majority of participants also believed that physicians should be aware of traditional medicines used by patients (120, 93.0%), most of whom strongly agreed (89, 69.0%). Finally, approximately three-quarters of participants believed that the clinical curriculum should incorporate a rotation in an Indigenous community (95, 73.6%), although less than one-half strongly agreed with this statement (55, 42.6%). (Table 2) The mean score of the factor formed by these variables was 3.48 (SD = 0.63).

#### ‘Interest in working in an Indigenous community’

While nearly one-half of participants expressed that they would consider working in an Indigenous community (60, 46.5%), more than one-quarter indicated that they would not (39, 30.3%). The remaining quarter (30, 23.3%) were uncertain. Among participants who provided a definitive answer, a greater proportion of females (35, 63.6%) answered favourably compared to males (24, 55.8%), although the difference was not statistically significant (χ^2^ = 0.616, *p* = 0.432). Interestingly, the proportion of participants who agreed with this statement increased steadily over the three years of study completion (43.3% in 2014, 58.9% in 2015, 80.0% in 2016), differences which were statistically significant (χ^2^ = 8.518, *p* = 0.014). (Table 2).

### Independent variables: (Tables 3 and 4)

Overall, participants scored highest on ‘*Knowledge of Indigenous health inequities’* (μ = 3.53, SD = 0.485), followed by ‘*Knowledge of colonization and links to Indigenous health inequities*’ (μ = 3.34, 0.606), and ‘*Sociopolitical attitudes about Indigenous peoples*’ (μ = 2.86, SD = 0.651), with higher attitude scores indicating more positive attitudes. *‘Self-rated educational preparedness to work with Indigenous patients’* had the lowest mean score (μ = 1.92) compared to other factors and the highest standard deviation (SD = 0.781), indicating most variation in this factor among participants.

**Table 3:** Results of Principal Components Analysis and descriptive statistics of items used to assess independent variables (*N* = 129)

	Component Loading ^b^	Mean (SD)	Strongly disagree N(%)	Somewhat disagree N(%)	Somewhat agree N(%)	Strongly agree N(%)	I don’t know N(%)
*Sociopolitical attitudes about Indigenous peoples (α = 0.777)* ^a^		2.86 (0.651)					
Canada’s Aboriginal people are treated well by the Canadian government ^c^	0.661	2.88 (0.869)	32 (24.8)	46 (35.7)	34 (26.4)	6 (4.7)	11 (8.5)
No additional taxpayer money should go to any Reserve until external auditors can be put in place to ensure financial accountability ^c^	0.761	2.39 (1.00)	15 (11.6)	32 (24.8)	30 (23.3)	23 (17.8)	29 (22.5)
Aboriginal protesters are conducting justified and legitimate protests by shutting down roads and rail lines going through their communities	0.708	2.42 (0.913)	16 (12.4)	33 (25.6)	33 (25.6)	11 (8.5)	36 (27.9)
Most Aboriginal people have been trying to get ahead economically at the expense of non-Aboriginal people ^c^	0.712	3.54 (0.633)	65 (50.4)	37 (28.7)	5 (3.9)	1 (0.8)	21 (16.3)
Now that the federal government has apologized for the residential school system, it is time for Aboriginal people to leave the past behind and move on ^c^	0.620	2.91 (0.937)	34 (26.4)	47 (36.4)	22 (17.1)	11 (8.5)	15(11.6)
Knowledge of colonization and its links to Indigenous health inequities (α = 0.865)^a^		3.34 (0.606)					
Loss of Aboriginal peoples’ traditional lifestyle is a result of colonization	0.784	3.28 (0.654)	1 (0.8)	9 (7.0)	57 (44.2)	42 (32.6)	20 (15.5)
Loss of Aboriginal peoples’ traditional lifestyle is a negative contributing factor to their health	0.718	3.25 (0.805)	2 (1.6)	18 (14.0)	37 (28.7)	49 (38.0)	23 (17.8)
The residential school system has caused negative health outcomes for Aboriginal peoples	0.688	3.50 (0.650)	1 (0.8)	6 (4.7)	38 (29.5)	62 (48.1)	22 (17.1)
Health effects of the residential school system are propagated through several generations	0.764	3.37 (0.783)	4 (3.1)	8 (6.2)	39 (30.2)	56 (43.4)	22 (17.1)
	*Component Loading* ^b^	*Mean (SD)*	*Strongly disagree N(%)*	*Some-what disagree N(%)*	*Somewhat agree N(%)*	*Strongly agree N(%)*	*I don’t know N(%)*
*Knowledge of Indigenous health inequities (α = 0.820)* ^a^		3.53 (0.485)					
Aboriginal peoples have a higher prevalence of alcohol abuse than the general population	0.723	3.54 (0.550)	0	3 (2.6)	48 (37.2)	66 (51.2)	12 (9.3)
Aboriginal peoples have a higher prevalence of obesity than the general population.	0.892	3.50 (0.624)	0	7 (5.4)	38 (29.5)	58 (45.0)	26 (20.2)
Aboriginal peoples have a higher prevalence of Type 2 diabetes than the general population.	0.888	3.51 (0.606)	1 (0.8)	3 (2.3)	42 (32.6)	59 (45.7)	24 (18.3)
Aboriginal peoples have a higher suicide rate than the general population.	0.682	3.70 (0.517)	1 (0.8)	28 (21.7)	0	76 (58.9)	24 (18.6)
*Self-rated educational preparedness to work with Indigenous patients* *(α = 0.816)* ^a^		1.92 (0.781)					
I have been adequately educated regarding social issues facing Aboriginal peoples.	0.924	1.81 (0.899)	56 (43.4)	44 (34.1)	16 (12.4)	8 (6.4)	5 (3.9)
I have been adequately educated regarding health issues facing Aboriginal peoples.	0.906	1.78 (0.867)	57 (44.2)	44 (34.1)	18 (14.0)	6 (4.7)	4 (3.1)
I anticipate that I will feel comfortable discussing trust issues with Aboriginal patients.	0.735	2.15 (0.962)	35 (27.1)	43 (33.3)	29 (22.5)	12 (9.3)	10 (7.8)

^a^ Cronbach’s alpha for the subscale

^b^ Component loadings < 0.4 suppressed

^c^ Items reverse coded so that strongly agree = lower attitude score; strongly disagree = higher attitude score

**Table 4:** Linear regression predicting perceived importance of learning about Indigenous health

*F* = 8.623, *p* < 0.0001, Adjusted *R*^*2*^ = 0.286			
Predictors	β	SE^a^	p
Sociopolitical Attitudes about Indigenous Peoples	0.277 **	0.090	0.003
Knowledge of Indigenous Health Inequities	−0.063	0.105	0.551
Knowledge of colonization and its links to Indigenous health inequities	0.290 **	0.097	0.005
Self-rated educational preparedness to work with Indigenous patients	−0.087	0.072	0.225
Age	0.161	0.099	0.105
Gender	−0.246 *	0.106	0.022

^a^ *SE* Standard Error

* = p < 0.05; ** = p < 0.01; *** = p < 0.001

#### ‘Sociopolitical attitudes about Indigenous peoples’

Participants generally disagreed with the concepts that Indigenous peoples were treated well by the government (78, 60.5%) and that Indigenous peoples should leave the past behind and move on (81, 62.8%). The majority of participants (102, 78.7%) also disagreed that Indigenous peoples are trying to get ahead economically at the expense of non-Indigenous people. However, responses were more divisive for other questions: only one-third (44, 34.1%) of the participants agreed that Indigenous protestors are conducting justified and legitimate protests by shutting down roads and rail lines going through their communities, and over one-third (53, 41.1%) agreed that no additional taxpayer money should go to any Reserve until external auditors can be put in place to ensure financial accountability.

#### ‘Knowledge of colonization and links to Indigenous health inequities’

The majority of participants agreed that the loss of Indigenous peoples’ traditional lifestyles was a result of colonization (99, 76.8%) and that this loss is a negative contributing factor to health outcomes (86, 66.7%). The majority of participants also agreed that the residential school system caused negative health outcomes for Indigenous peoples (100, 77.6%), which were propagated through several generations (95, 73.6%).

#### ‘Knowledge of Indigenous health inequities’

The majority of participants were aware of the most common health inequities faced by Indigenous peoples; 101 participants (78.3%) agreed that Indigenous peoples face a higher rate of diabetes and 96 (74.5%) agreed that Indigenous peoples face a higher rate of obesity. More than half of participants also believed that the suicide rate is higher among Indigenous peoples (76, 58.9%) as well as alcohol abuse (114, 88.4%).

#### ‘Self-rated educational preparedness to work with Indigenous patients’

Despite participants’ perceptions of the importance of learning about Indigenous health, over three-quarters did not believe themselves to be adequately educated on these topics: 100 (77.5%) participants disagreed that they have been adequately educated on social issues, and 101 (78.3%) disagreed that they have been adequately educated on health issues. Indeed, nearly one-half of participants strongly disagreed with these statements (43.4 and 44.2% for social and health issues, respectively). When asked if they anticipate that they will “*feel comfortable discussing trust issues with Indigenous patients*”, the majority of participants (78, 60.4%) disagreed.

### Multivariate analyses

Individual multivariate models were constructed for each outcome using the independent variables defined by the PCA (*‘Sociopolitical attitudes towards Indigenous peoples’; ‘Knowledge of colonization and its links to health inequities’; ‘Knowledge of Indigenous health inequities’; ‘Self-rated educational preparedness to work with Indigenous patients’*) and controlled for age and gender.

The first multivariate model used linear regression to model *‘Perceived importance of Indigenous content in curricula’*. Age, gender, and the four independent variables accounted for 28.6% of the variance in the outcome (R^2^ = 0.286, F(6,108) = 8.623, *p* < 0.0001). Two of the independent variables were statistically significant: *‘Sociopolitical attitudes towards Indigenous peoples’* (β = 0.277, *p* = 0.003) was the strongest, followed by *‘Knowledge of colonization and links to health inequities’* (β = 0.290, *p* = 0.005). Gender was also statistically significant (β = − 0.246, *p* = 0.022), indicating that female gender was positively associated with higher perceived importance scores. Age was not significant (β = 0.161, *p* = 0.105). (Table 5).

**Table 5:** Logistic regression predicting interest in working in an Indigenous community

Variable	B	Odds Ratio	p
Constant	−0.332	0.718	0.915
Sociopolitical Attitudes about Indigenous Peoples	2.310***	10.076	< 0.001
Knowledge of Indigenous Health Inequities	−2.038**	0.130	0.003
Knowledge of colonization and its links to Indigenous health inequities	0.619	1.858	0.257
Self-rated educational preparedness to work with Indigenous patients	0.206	1.229	0.612
Age	0.506	1.659	0.363
Gender	−0.953	0.386	0.122
Nagelkerke pseudo r-square Chi-square	48.9% 40.99, df = 6, *p* < 0.0001		

* = p < 0.05; ** = p < 0.01; *** = p < 0.001

Second, *‘Interest in working in an Indigenous community*’ (DV) was modelled using logistic regression with a dichotomized outcome (agree/disagree). The full model indicated that the independent variables significantly predicted more variance than the constant-only model (χ^2^ = 40.999, df = 6, *p* < 0.0001). Of the independent variables, the Wald criterion demonstrated that *‘Sociopolitical attitudes towards Indigenous peoples’* (β = 2.310, *p* < 0.001) was the strongest (and only) positive predictor. *‘Knowledge of Indigenous health inequities’* (β = − 2.038, *p* = 0.003) was a significant negative predictor of the outcome; increasing scores on *‘Knowledge of Indigenous health inequities’* was associated with decreasing interest in working in an Indigenous community. Neither age nor gender were statistically significant (Table 5).

## Discussion

The results of this study suggest that the first-year medical students in our sample believe that learning about Indigenous peoples is important, but most do not feel adequately educated or prepared to work with Indigenous populations. This finding is consistent with past studies like that of Zhou et al., Larson et al., and Jumah et al., who documented similar interest in working in Indigenous communities among medical students and medical residents at other academic institutions at later stages of their education that was coupled with significant doubt about their levels of preparedness to do so. [11, 19, 20] This study also introduces the finding that medical students are strongly interested in learning more about Indigenous health and are particularly interested in experiential learning, such as clinical rotations in Indigenous communities. Together, these results support two conclusions: first, Indigenous health teaching is a necessary part of medical education, as students entering the program do not possess adequate background knowledge in these domains. This finding has been established and reiterated in past studies that have highlighted the fundamental lack of education about Indigenous realities at all levels of education, including elementary and secondary school. [9, 30, 31] Second, there is a general need across Canadian medical schools for greater focus on practical skills in working with Indigenous patients that will increase medical student preparedness and confidence in navigating real-life clinical scenarios. Medical schools must consider more comprehensive approaches to teaching about Indigenous health that move beyond didactic teaching to include experiential learning and interactive, longitudinal teaching in authentic environments, which are recommendations that have been made for cultural safety training more generally. [10, 23, 32] Currently, integration of such approaches is rare, despite strong recommendations for doing so by the Association of Faculties of Medicine of Canada Council of Deans, Aboriginal Health Working Group, and the Canadian Federation of Medical Students [33–35].

This study also clearly indicates that positive sociopolitical attitudes about Indigenous peoples is significantly associated with perceived importance of learning about Indigenous health, as well as interest in working in Indigenous communities. The importance of attitudes in shaping and defining behaviours has been conceptualized as ‘attitude-behaviour consistency’ in psychological theory, with most recent research postulating that though the relationship is not necessarily linear nor causal, positive attitudes are important predictors of patterns of behavior towards minority groups, including voting patterns, charitable donations, and verbal behaviors. [36–40] In the context of health care, changing medical student attitudes has already been identified as an important means of improving care for other unique populations, including patients with substance use disorders, disabilities, and older adults. [41–43] The need to address attitudes in medical school curricula is further reinforced by studies that identify the pragmatic utility of fostering positive attitudes in students: positive attitudes are a significant predictor of preparedness to work in contexts requiring different skills, such as mental health care, rural medical practice, and within Indigenous Australian communities. [16–18] Given this, addressing student attitudes must be a priority and curriculum must be re-oriented to challenge students to confront their implicit biases, beliefs, power, and privilege, all of which contribute to attitude development and are inherent to the original conceptual formulation of cultural safety. [44]

In addition to sociopolitical attitudes, knowledge of colonization and its links to Indigenous health inequities was associated with increased perceived importance of learning Indigenous health content in medical school. Not unexpectedly, it is logically congruent that those who enter medical school without this knowledge are also the ones who perceive it as less important and will likely be in particular need of educational approaches that can target and change attitudes. Knowledge of various aspects of colonization in Canada and its effects on the health and well-being of today’s Indigenous peoples is still not well known among the general public and has not been comprehensively included in elementary and secondary school curricula in Canada, which would have been undertaken by many current medical students. [30, 31] Given this, medical school curricula must begin teaching about Indigenous health by providing students with contextual information on Canada’s colonial history prior to providing health-related knowledge and training on competencies and skills necessary to providing care for Indigenous peoples. Indeed, such a curriculum will likely take more time than what is currently allocated to Indigenous health teaching; for the students in our sample, roughly only ten hours of Indigenous health content are included across all four years of medical school.

It is important to note that medical school influences the attitudes of students not only through formal curricula, but also through informal, ‘hidden’ curricula that exists insidiously in the organizational structure and culture of the institution and its leadership. [45, 46] These implicit aspects of the practice of medicine, as learned through the lack of focus on certain issues in the curricula or through teachers’ and mentors’ offhand comments or actions, for example, have proven to be as important or perhaps even more influential than taught curriculum. [45, 46] Ly and Crowshoe [12] suggest that teachers’ impressions of Indigenous peoples have a direct impact on students’ perceptions. Thus, although the sociopolitical attitudes of first-year medical students towards Indigenous peoples in our sample appear relatively empathetic, the four-year curriculum of medical education still plays a critical role in reinforcing (or not) these attitudes. As students progress through medical school, their moral reasoning and attitude scores decline, possibly as a result of increasing cynicism, loss of idealism, and the impact of the hidden curriculum. [47, 48] Thus, the degree to which students may positively regard Indigenous peoples in their first year of studies, as is illustrated by our results, cannot be considered representative of their ultimate beliefs; attitudes may be transient and medical schools must be aware of the power they have to solidify or change attitudes. By extension, medical schools must also assume responsibility for similarly cultivating respectful sociopolitical attitudes among medical educators so that cultural safety is not simply a concept explained to students, but tangibly observed in the words and actions of their preceptors.

Contrary to our expectations, however, knowledge of Indigenous health inequities was significantly negatively associated with interest in working in an Indigenous community. There are many potential explanations for this: students with less knowledge of Indigenous health may feel greater impetus to fill their knowledge gap by working in an Indigenous community. This hypothesis is supported by the fact that the majority of participants in our sample possessed positive attitudes towards Indigenous peoples; for those with little knowledge, these positive attitudes may be a driver to learn more through work experience. Additionally, students who know more about Indigenous health inequities may have a greater appreciation of the complexity and difficulty of clinical practice in Indigenous communities, which is often impacted heavily by political and economic factors that dictate, for example, access to clean water, stable food supplies, and living-wage incomes. Given that these elements are beyond the immediate control of health professionals, medical students who understand the difficulty of the realities in many Indigenous communities may feel unprepared – emotionally, mentally, and with respect to their skill set – to practice in such challenging and isolating environments. Indeed, this finding may also be reflective of the disparaging and disillusioning ways in which work within Indigenous communities is portrayed within medical school. Regardless of the origins of this finding, its implications are aligned with our conclusion that delivery of knowledge is generally inadequate in preparing students to work in Indigenous contexts; there is a need to target the development of practical skills that enable students to have the confidence and the courage to interact and to serve Indigenous patients in meaningful and effective ways.

### Strengths and limitations

The repetition of this study among three cohorts of students strengthens its conclusions by increasing the sample size and the internal validity of results for local curriculum development. Response rates among each cohort were a limitation; they did not exceed 50% for any given cohort, despite many efforts to encourage higher participation. Finally, our sample was limited to students from one medical school, and although this student body consists of individuals from many different provinces, their exposure to Indigenous peoples and education may still differ from that of medical students who attend school elsewhere in the country. Although this is an inherent limitation to the case study design, the thorough examination of students in a single context is arguably more ideal for exploratory investigation in this novel area of study and also provides an exemplar for future work in local curriculum development that is not centrally governed in Canada, but operates at the level of individual institutions. [49]

## Conclusion

In this case study, we have demonstrated that even at the onset of their medical education, medical students believe learning about Indigenous health is important and they are interested in future work in Indigenous communities, but feel that they would not be prepared to work with Indigenous peoples given their current knowledge and experiences. Furthermore, we demonstrate that interest in learning about and working within Indigenous communities is most strongly associated with positive attitudes about Indigenous peoples *in conjunction* with knowledge about colonization and its links with health inequities. These findings highlight the need for our medical schools to support Indigenous health curriculum that is comprehensive and moves beyond the current provision of formal learning to also include clinical rotations and placements with Indigenous patients and assessment of the hidden curriculum as delivered through the institutional environment and training and recruitment of faculty. Given the limited progress on these objectives within the medical education landscape more generally, we recognize that this challenge is not limited to our context and we extend this recommendation as a collective goal to academic institutions at every level of training. Ultimately, changes to medical education are important in the provision of more culturally-relevant and safe care to Indigenous peoples and to the overall reduction of the systemic injustices against Indigenous peoples that are present in the Canadian health care system today.

## Additional files

Additional file 1: Survey instrument. (PDF 721 kb)

## Footnotes

1 Settler people are ‘peoples who occupy lands previously stolen or in the process of being stolen from their Indigenous inhabitants, or who are otherwise members of ‘Settler society’ founded on co-opted lands and resources.’ (Barker, 2006, pg22) [50]

2 Participants were permitted to select more than one ethnic affiliation in the survey question
