# Supplementary material for: Predictors of medical student interest in Indigenous health learning and clinical practice: a Canadian case study
Source: BMC Med Educ. 2018 Dec 14;18:307. doi: 10.1186/s12909-018-1401-1 (PMC6295008; doi:10.1186/s12909-018-1401-1)
Supplement: Supplementary file 1 — Survey instrument. (PDF 721 kb) [file 12909_2018_1401_MOESM1_ESM.pdf]

"You go to the emergency, you're treated like dirt": Reducing Aboriginal experiences of racism in health care through a medical school intervention
A Survey

Survey questions have been adapted from the following sources: Denis (2011)[1], IPSOS-Reid (2012)[2], Environics Institute (2010)[3], and Zhou et al. (2011)[4]

This survey should take approximately 20–25 minutes to complete.
Please check the appropriate answer.

SECTION A. Demographics

A1. Age

☐ 18–24 ☐ 25–34 ☐ 35–44 ☐ 45–54 ☐ 55–64 ☐ 65+ ☐ Prefer to Leave Blank

A2. Ethnic Origin

☐ Canadian	☐ English	☐ French	☐ Chinese	☐ East Indian
☐ Italian	☐ German	☐ Scottish	☐ Irish	☐ Cree
☐ Mi'kmaq	☐ Salish	☐ Métis	☐ Inuit	☐ First Nations
☐ Filipino	☐ Dutch	☐ Ukrainian	☐ Polish	☐ Portuguese
☐ Greek	☐ Korean	☐ Vietnamese	☐ Jamaican	☐ Jewish
☐ Lebanese	☐ Salvadorean	☐ Somali	☐ Colombian	☐ Other
☐ Prefer to Leave Blank				

A3. Sex

☐ Female ☐ Male ☐ Transgendered ☐ Other ☐ Prefer to Leave Blank

A4. Which is your home campus?

☐ Halifax ☐ Saint John

1 Denis, J. 2011. Canadian apartheid: Boundaries and bridges in Aboriginal-White relations. PhD Dissertation;

2 IPSOS-Reid online omnibus survey, 2012;

3 Environics Institute. 2010. Urban Aboriginal Peoples Study;

4 Zhou, A.W., et al. 2011. Efficacy of a 3-hour Aboriginal health teaching in the medical curriculum: Are we changing student knowledge and attitudes? *Health Education Journal,* 71(2), 180-188.

SECTION B.

Please answer by checking either True, False, or I Don't Know:

B1. Most Reserves are far from urban centres

☐ True ◯ False ◯ I Don't Know

B2. Each Reserve has a health care team in place to address the needs of its members

☐ True ☐ False ☐ I Don't Know

B3. The Aboriginal population rarely needs to travel to an urban centre for health care needs

☐ True ☐ False ☐ I Don't Know

B4. The medicine wheel is a holistic approach to medicine

☐ True ☐ False ☐ I Don't Know

SECTION C.

For the following questions, please check the item that best fits your opinion.

C1. How would you rate the Canadian System of Primary and Secondary Education with respect to teaching students about Canada's Aboriginal people, their pre-contact, early contact, and late contact history with the Settler (primarily English and French) population?

☐ Excellent ☐ Good ☐ Fair ☐ Poor ☐ I Don't Know

C2. Which statement best represents your thoughts about Aboriginal people?

☐ Have unique rights/privileges as first inhabitants of Canada OR

☐ Just like any other cultural/ethnic group in Canada OR

☐ I Don't Know

Do you strongly approve, somewhat approve, somewhat disapprove, or strongly disapprove of how each of the following have handled First Nation issues?

C3. The Idle No More movement

☐ Strongly approve ☐ Somewhat approve ☐ Somewhat disapprove ☐ Strongly disapprove ☐ I Don't Know

C4. Anti-fracking protestors at Elsipogtog First Nation

☐ Strongly approve ☐ Somewhat approve ☐ Somewhat disapprove ☐ Strongly disapprove ☐ I Don't Know

C5. The RCMP

☐ Strongly approve ☐ Somewhat approve ☐ Somewhat disapprove ☐ Strongly disapprove ☐ I Don't Know

C6. The federal government

☐ Strongly approve ☐ Somewhat approve ☐ Somewhat disapprove ☐ Strongly disapprove ☐ I Don't Know

SECTION D.

For the following questions, please check the item that best fits your opinion.
Do you strongly agree, somewhat agree, somewhat disagree, or strongly disagree with each of the following statements:

D1. Canada's Aboriginal peoples are treated well by the Canadian government.

☐ Strongly disagree ☐ Somewhat disagree ☐ Somewhat agree ☐ Strongly agree ☐ I Don't Know

D2. Much of the money spent by the federal government for on-reserve services is managed well by the **Aboriginal** leaders and communities who receive it.

☐ Strongly disagree ☐ Somewhat disagree ☐ Somewhat agree ☐ Strongly agree ☐ I Don't Know

D3. No additional taxpayer money should go to any Reserve until external auditors can be put in place to ensure financial accountability.

☐ Strongly disagree ☐ Somewhat disagree ☐ Somewhat agree ☐ Strongly agree ☐ I Don't Know

D4. Most of the problems of **Aboriginal people are brought on by themselves.**

☐ Strongly disagree ☐ Somewhat disagree ☐ Somewhat agree ☐ Strongly agree ☐ I Don't Know

D5. Aboriginal protestors are conducting justified and legitimate protests by shutting down roads and rail lines going through their communities.

☐ Strongly disagree ☐ Somewhat disagree ☐ Somewhat agree ☐ Strongly agree ☐ I Don't Know

D6. Court rulings that protect the rights of Aboriginal people usually hurt the rights of non-Aboriginal people.

☐ Strongly disagree ☐ Somewhat disagree ☐ Somewhat agree ☐ Strongly agree ☐ I Don't Know

D7. Aboriginal people in Canada share many basic values and goals with non-Aboriginal people in Canada.

☐ Strongly disagree ☐ Somewhat disagree ☐ Somewhat agree ☐ Strongly agree ☐ I Don't Know

D8. Aboriginal people have too little influence when it comes to enforcing treaty rights.

☐ Strongly disagree ☐ Somewhat disagree ☐ Somewhat agree ☐ Strongly agree ☐ I Don't Know

D9. Most Aboriginal peoples have been trying to get ahead economically at the expense of non-Aboriginal peoples.

☐ Strongly disagree ☐ Somewhat disagree ☐ Somewhat agree ☐ Strongly agree ☐ I Don't Know

D10. The federal government is spending too much on improving the living conditions of Aboriginal peoples.

☐ Strongly disagree ☐ Somewhat disagree ☐ Somewhat agree ☐ Strongly agree ☐ I Don't Know

D11. Now that the federal government has apologized for the residential school system, it is time for Aboriginal people to leave the past behind and move on.

☐ Strongly disagree ☐ Somewhat disagree ☐ Somewhat agree ☐ Strongly agree ☐ I Don't Know

D12. The 2008 residential school apology was a first step, but now it is time for the government to show it is serious about healing and reconciliation.

☐ Strongly disagree ☐ Somewhat disagree ☐ Somewhat agree ☐ Strongly agree ☐ I Don't Know

D13. The Aboriginal population in Canada has equal access to government provided health care.

☐ Strongly disagree ☐ Somewhat disagree ☐ Somewhat agree ☐ Strongly agree ☐ I Don't Know

D14. Many members of the Aboriginal population rely on their traditional medicines and ceremonies in everyday life.

☐ Strongly disagree ☐ Somewhat disagree ☐ Somewhat agree ☐ Strongly agree ☐ I Don't Know

D15. Aboriginal peoples frequently use tobacco for traditional purposes.

☐ Strongly disagree ☐ Somewhat disagree ☐ Somewhat agree ☐ Strongly agree ☐ I Don't Know

D16. Aboriginal peoples frequently use alcohol for traditional purposes.

- [ ] Strongly disagree
- [ ] Somewhat disagree
- [ ] Somewhat agree
- [ ] Strongly agree
- [ ] I Don't Know

D17. Aboriginal peoples have a higher prevalence of alcohol abuse than the general population.

- [ ] Strongly disagree
- [ ] Somewhat disagree
- [ ] Somewhat agree
- [ ] Strongly agree
- [ ] I Don't Know

D18. Aboriginal peoples have a higher prevalence of obesity than the general population.

- [ ] Strongly disagree
- [ ] Somewhat disagree
- [ ] Somewhat agree
- [ ] Strongly agree
- [ ] I Don't Know

D19. Aboriginal peoples have a higher prevalence of Type 2 diabetes than the general population.

- [ ] Strongly disagree
- [ ] Somewhat disagree
- [ ] Somewhat agree
- [ ] Strongly agree
- [ ] I Don't Know

D20. Aboriginal peoples have a higher suicide rate than the general population.

- [ ] Strongly disagree
- [ ] Somewhat disagree
- [ ] Somewhat agree
- [ ] Strongly agree
- [ ] I Don't Know

D21. Conventional (i.e. western) physicians should be aware of traditional medicine(s) that their patients may be using.

- [ ] Strongly disagree
- [ ] Somewhat disagree
- [ ] Somewhat agree
- [ ] Strongly agree
- [ ] I Don't Know

D22. Aboriginal peoples are more likely to comply with a treatment plan than the general Canadian population.

- [ ] Strongly disagree
- [ ] Somewhat disagree
- [ ] Somewhat agree
- [ ] Strongly agree
- [ ] I Don't Know

D23. Aboriginal peoples are generally mistrusting of conventional (i.e. western) physicians.

- [ ] Strongly disagree
- [ ] Somewhat disagree
- [ ] Somewhat agree
- [ ] Strongly agree
- [ ] I Don't Know

D24. Aboriginal peoples do not communicate as openly with conventional physicians as compared to the general population.

- [ ] Strongly disagree
- [ ] Somewhat disagree
- [ ] Somewhat agree
- [ ] Strongly agree
- [ ] I Don't Know

D25. Loss of Aboriginal peoples' traditional lifestyle is a result of colonization.

- [ ] Strongly disagree
- [ ] Somewhat disagree
- [ ] Somewhat agree
- [ ] Strongly agree
- [ ] I Don't Know

D26. Loss of Aboriginal peoples' traditional lifestyle is a negative contributing factor to their health.

- [ ] Strongly disagree
- [ ] Somewhat disagree
- [ ] Somewhat agree
- [ ] Strongly agree
- [ ] I Don't Know

D27. The residential school system has caused negative health outcomes for Aboriginal peoples.

☐ Strongly disagree ☐ Somewhat disagree ☐ Somewhat agree ☐ Strongly agree ☐ I Don't Know

D28. Health effects of the residential school system are propagated through several generations.

☐ Strongly disagree ☐ Somewhat disagree ☐ Somewhat agree ☐ Strongly agree ☐ I Don't Know

D29. I have been adequately educated regarding social issues facing Aboriginal peoples.

☐ Strongly disagree ☐ Somewhat disagree ☐ Somewhat agree ☐ Strongly agree ☐ I Don't Know

D30. I have been adequately educated regarding health issues facing Aboriginal peoples.

☐ Strongly disagree ☐ Somewhat disagree ☐ Somewhat agree ☐ Strongly agree ☐ I Don't Know

D31. I anticipate that I will feel comfortable discussing trust issues with Aboriginal patients.

☐ Strongly disagree ☐ Somewhat disagree ☐ Somewhat agree ☐ Strongly agree ☐ I Don't Know

D32. It is valuable to me as a physician to be educated on social issues concerning Aboriginal peoples.

☐ Strongly disagree ☐ Somewhat disagree ☐ Somewhat agree ☐ Strongly agree ☐ I Don't Know

D33. It is valuable to me as a physician to be educated on the health issues concerning Aboriginal peoples.

☐ Strongly disagree ☐ Somewhat disagree ☐ Somewhat agree ☐ Strongly agree ☐ I Don't Know

D34. The clinical curriculum should incorporate a rotation in a First Nations, Metis and/or Inuit community.

☐ Strongly disagree ☐ Somewhat disagree ☐ Somewhat agree ☐ Strongly agree ☐ I Don't Know

D35. I would consider working in a First Nations, Metis and/or Inuit community.

☐ Strongly disagree ☐ Somewhat disagree ☐ Somewhat agree ☐ Strongly agree ☐ I Don't Know

Thank you for your participation.
